# Association of child weight with attendance at a healthy lifestyle service among women with obesity during pregnancy

**DOI:** 10.1111/mcn.13629

**Published:** 2024-02-04

**Authors:** Frankie J. Fair, Hora Soltani

**Affiliations:** ^1^ College of Health, Wellbeing and Life Sciences Sheffield Hallam University Sheffield UK

**Keywords:** childhood obesity, developmental origins of disease, gestational weight gain, healthy lifestyle, maternal obesity

## Abstract

Women with obesity during pregnancy are at increased risk of excessive gestational weight gain (GWG) and other maternal and infant adverse outcomes, which all potentially increase childhood obesity. This study explored infant weight outcomes for women with a body mass index (BMI) ≥ 35 kg/m² who were offered an antenatal healthy lifestyle service. A retrospective cohort study, including linking data from two separate health care Trusts, was undertaken. Data were collected from maternity records for women with a BMI ≥ 35 kg/m^2^ referred to an antenatal healthy lifestyle service from 2009 to 2015. The respective child's weight outcome data was additionally collected from health and National Child Measurement Programme records. Univariate logistic regression determined the odds of childhood overweight, obesity and severe obesity according to attendance at the antenatal healthy lifestyle service, GWG and sociodemographic characteristics. Factors significant (*p* < 0.05) within the univariate analysis were entered into multiple logistic regression models. Among women with a BMI ≥ 35 kg/m², 30.4% of their children were obese at school entry and 13.3% severely obese. Healthy lifestyle service attendance was not associated with childhood overweight or obesity at any point within the univariate analysis. At school age multiple regression analysis showed the odds of overweight and obesity increased with excessive GWG and the odds of obesity decreased with a parent in a professional occupation, additionally having a mother who smoked in pregnancy increased severe obesity. Women should be supported to optimise their BMI before pregnancy. Additionally, rather than exclusively focusing on changing an individual's behaviour, future interventions should consider external influences such as the woman's family, friends and sociodemographic background.

## Key messages

- Among children born to women with a body mass index (BMI) ≥ 35 kg/m^2^ almost 50% were classified as overweight or obese at age 5. Of these, only 15.6% had been born large for gestational age.
- This brief antenatal healthy lifestyle intervention provided to mothers with obesity did not significantly reduce child's overweight or obesity.
- Demographic factors such as household occupation and maternal smoking during pregnancy were associated with long‐term childhood obesity.
- More emphasis is required on interventions that support women to optimise their BMI before pregnancy.
- Future interventions should consider external influences on the woman for example through a socioecological framework.

## Graphical abstract

Among children born to women with a body mass index (BMI) ≥ 35 kg/m^2^ almost 50% were classified as overweight or obese at age 5. The mother attending a brief antenatal healthy lifestyle intervention did not reduce child obesity. Demographic factors including household occupation and maternal smoking during pregnancy were associated with long‐term childhood obesity.

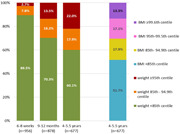

## 1 INTRODUCTION

In the United Kingdom, 32.5% of women are classified as overweight (body mass index [BMI]: 25–29.9 kg/m²) and a further 26.4% as obese (BMI ≥ 30 kg/m²) (Baker, 2023). Women affected by overweight or obesity account for over 50% of maternities, and 22.2% of pregnancies in the UK were in women with obesity in 2018–2019 (National Health Service National Health Service [NHS] Digital, 2019a).

Women with overweight or obesity before pregnancy are at high risk of excessive gestational weight gain (GWG) (Samura et al., 2016). Obesity during pregnancy and excessive GWG are both associated with an increased risk of adverse outcomes for both the mother and the infant. Adverse outcomes for the mother include increased risk of gestational diabetes (Najafi et al., 2019; Santos et al., 2019), pre‐eclampsia (He et al., 2020; Santos et al., 2019), preterm birth (Santos et al., 2019) and caesarean birth (Goldstein et al., 2017; Kim et al., 2016). The adverse outcomes for the infant include an increased risk of being large for gestational age (LGA) (Goldstein et al., 2017; Santos et al., 2019; Shin & Song, 2015), and poorer breastfeeding outcomes (Huang et al., 2019). Additionally, maternal obesity and excessive GWG have been associated with increased childhood obesity (Voerman et al., 2019), as have many of their associated adverse outcomes such as gestational diabetes, hypertension, reduced breastfeeding (Skrypnik et al., 2019) and caesarean birth (Masukume et al., 2019). Overall, the proportion of overweight or obesity in early childhood (2–5 years) estimated to be attributable to maternal prepregnant obesity and excessive GWG is 10.2% and 11.4%, respectively (Voerman et al., 2019). Rates of childhood obesity by age 7 have been shown to vary across Europe, with the lowest prevalence in Denmark (5.7%) and the highest in Greece (17.1%) (WHO European Region, 2022). In England, the prevalence of obesity in children when starting school (age 4–5 years) was 10.1% in 2021–2022 (NHS Digital, 2022).

Pregnancy has been suggested as a good opportunity to influence behaviour change in mothers and their families through adaptations to lifestyle such as healthy eating, physical activity and weight management (Phelan, 2010). However, a recent UK survey found maternal healthy lifestyle service provision for women with obesity to be inconsistent in availability, BMI eligibility criteria and content (Fair et al., 2020). Additionally, interviews with providers and commissioners alongside the above survey also identified uncertainty among professionals about what constitutes the most suitable service to tackle maternal obesity (Fair et al., 2020). Antenatal lifestyle interventions, mainly focussed on healthy eating and physical activity, have been evaluated within numerous studies and systematic reviews for their impact on maternal outcomes such as GWG and mode of birth, as well as neonatal outcomes such as birthweight and gestational age at birth (Dodd et al., 2014; Fair & Soltani, 2021; Hill et al., 2013; Thangaratinam et al., 2012). However, little has been done to date to evaluate pregnancy lifestyle interventions on longer term infant health. This is despite the impact of maternal health and diet before and during pregnancy being increasingly understood on long‐term offspring health and development, through the role of epigenetics (Aldhous et al., 2018; Lorite Mingot et al., 2017). Within two systematic reviews of lifestyle interventions during pregnancy (Dalrymple et al., 2018; Raab et al., 2021) few studies were found that evaluated childhood anthropometric outcomes up to school entry. Evidence around the effect of antenatal lifestyle interventions on long‐term child obesity is especially limited among women with obesity. This is despite these infants being recognised to be at increased risk of childhood obesity (Voerman et al., 2019). The need for studies which explore longer term health outcomes for mothers and infants of interventions in pregnancy has been recognised (Goldstein et al., 2016).

Socioeconomic inequalities are known to be strongly associated with the prevalence of obesity (Nguyen et al., 2023). Obesity is higher among those with the highest levels of deprivation, and food insecurity, those from an ethnic minority (Nguyen et al., 2023), as well as those with lower educational attainment (Devlieger et al., 2016). These factors therefore need careful consideration when exploring child's weight status. This study therefore aimed to explore the association between child overweight and obesity and attendance at an antenatal healthy lifestyle service intervention, along with other sociodemographic characteristics, for women with a BMI ≥ 35 kg/m² when booking for pregnancy care within one hospital Trust.

## 2 METHODS

### 2.1 Setting

In England, the NHS provides routine care to all pregnant women. Non‐NHS care is rare, with only 0.5% of all births in England and Wales in 2021 taking place in non‐NHS establishments or ‘elsewhere’ (Office for National Statistics [ONS], 2023). In July 2009, a midwife‐led antenatal healthy lifestyle service was established at Doncaster and Bassetlaw Teaching Hospitals NHS Foundation Trust which is within the Yorkshire and Humber region of England. This service was established in response to the recognition of high rates of maternal obesity within the local area. When established, the service offered a low‐intensity intervention to pregnant women with a BMI ≥ 35 kg/m^2^ at their first antenatal appointment which incorporated a visit at 16 weeks of gestation, with additional follow‐up visits available if the woman wanted them. In July 2012 service provision intensified, offering women routine appointments at 16, 28 and 36 gestational weeks. Due to service demands the provision at this point became exclusively for women with a BMI ≥ 40 kg/m². Midwives ran the service alongside other professionals such as dieticians and exercise programme providers, with specialised input from obstetricians and anaesthetists. Women were provided with support and advice around weight management; particularly minimising GWG, healthy eating, undertaking physical activity and breastfeeding. The aim of the clinic was to encourage and support women to make positive lifestyle choices and behavioural changes during pregnancy to optimise GWG and improve birth outcomes. The intention was that these changes could also be sustained after the birth. For example, women were encouraged to identify personal goals such as swapping an unhealthy food for a healthier one. Given that the healthy lifestyle service intervention commenced in 2009 it was possible to evaluate whether it was feasible to determine the association between pregnancy weight gain and antenatal healthy lifestyle service attendance on the rate of childhood obesity up to school age (4–5.5 years). This was done through the linkage of maternity records with health visitors' and National Child Measurement Programme records of infants' weight at 6–8 weeks, 9–12 months and school entry (4–5.5 years of age).

### 2.2 Data collection

Data were collected from hospital records for all women with a BMI ≥ 35 kg/m² who were offered the antenatal healthy lifestyle service between 2009 and 2015. Data extracted from these records included attendance at the antenatal healthy lifestyle service, maternal sociodemographic data and GWG, as well as pregnancy data including complications such as gestational diabetes, mode of birth and post‐natal data around infant feeding methods. Within the UK basic neonatal data, as well as the child's NHS number, are also stored within the maternity hospital records.

Within England, children are routinely weighed by health visitors at 6–8 weeks and 9–12 months. They are also weighed and measured by school nurses when starting school (age 4–5.5 years) as part of the National Child Measurement Programme. Health visitor and school nurse data are entered into the IT system; SystmOne. Data were collected from this database for infants born to women within the above cohort of women (attending antenatal care at the NHS Trust with the antenatal healthy lifestyle service between 2009 and 2015 with a BMI ≥ 35 kg/m^2^). Within both the hospital records and the SystmOne data, the child NHS number was pseudoanonymised to allow for data linkage. The MD5 hash system was used to pseudonymise the data. This takes a string of any length and encodes it into a 32‐character long ‘hash’. Upon entering the same string, the MD5 hash produced will always be the same. However, it is not possible to take the MD5 hash and convert it back to the original string. The NHS Trust data was therefore matched to data from SystmOne by pairng the MD5 hash code for the child's NHS number within the two datasets.

#### 2.2.1 Standard measures

BMI was calculated from weight at the first antenatal appointment using the formula weight/height squared (kg/m^2^). GWG was measured by subtracting weight at the first antenatal appointment from the final weight measured during pregnancy from the middle of the third trimester (34 + 0 weeks') gestation onwards. According to Institute of Medicine (IOM, 2009) guidance, the recommended weight gain for women with obesity is between 5 and 9 kg. In accordance with this recommendation, women were classified as gaining too little weight if they gained less than 5 kg, or as gaining excessive weight if their GWG was above the 9 kg recommended. Infant birthweight centiles were calculated using GROW charts (UK version 8.0.6.1) (Gardosi et al., 2011, 2020). These centiles are customised according to maternal height, maternal weight, ethnicity, parity, gestation and infant gender. This has been shown to be more accurate in populations with overweight and obesity (Pritchard et al., 2020). Birthweight above the 90th centile for gestational age was classified as LGA. Gestational diabetes was defined as fasting blood glucose ≥ 5.3 mmmol/L or blood glucose 2 h post 75 g glucose challenge ≥ 8.5 mmol/L.

Child weight percentiles at 6–8 weeks, 9–12 months and at school entry, as well as BMI percentiles at school entry, were calculated using the World Health Organization (WHO) Anthro (WHO, 2010) and AntroPlus (WHO, 2009) software. Children were classified as ‘overweight’ if their weight or BMI centile was between the 85th and 94.9th centile or as ‘obese’ if their weight or BMI centile was ≥95th centile. Additionally, children with severe obesity at school entry were identified as those with a BMI ≥ 99.6th centile. These classifications were in accordance with those used by the Office for Health Improvement and Disparities (OHID) (OHID, 2022a).

Occupation data was collected from women when first attending for pregnancy care. The woman and their partners' occupations were coded using the three‐category National Statistics Socio‐economic Classification system (ONS, 2010). The highest occupation category for each household (either for the woman or her partner) was utilised within the analysis. The Index of Multiple Deprivation (IMD) was used to measure deprivation, as this is the official measure of relative deprivation in England. The score for each area combines information from seven domains of deprivation (income, employment, education, health, crime, housing and living environment) to give one overall deprivation score from one (most deprived) to 32844 (least deprived) (Smith et al., 2015). These scores were designated into evenly sized quintiles. Quintile 1 included IMD scores 1–6568 and was the most deprived, quintile 2 included IMD scores 6569–13,137, quintile 3 included IMD scores 13,138–19,706, quintile 4 included IMD scores 19,707–26,275 and quintile 5 included IMD scores 26,276–32,844 which was the least deprived quintile. Due to the limited number of cases, the least deprived quintiles (quintiles 4 and 5) were then combined within the analysis.

### 2.3 Data analysis

Analysis was undertaken using SPSS 26.0. Univariate logistic regression was used to assess the association between childhood overweight, obesity and severe obesity according to uptake of the antenatal healthy lifestyle service and sociodemographic characteristics. GWG was the primary intended outcome of the antenatal healthy lifestyle service, it was therefore also assessed. Given their links with child obesity within the literature, gestational diabetes, hypertension, breastfeeding and caesarean birth were additionally evaluated to determine if they would require adjustment within the multiple logistic regression models. Odds ratios (OR) and 95% confidence intervals (95% CI) were calculated.

Any factors that were significant (*p* < 0.05) within the univariate analysis at any timepoint were included in the multiple logistic regression main effects model to determine the significance of each variable once controlling for other factors. Separate models were developed for childhood overweight or obesity at each of the timepoints 6–8 weeks, 9–12 months and at school entry, as well as severe obesity at school entry. These models were adjusted for anthropometric measures including maternal weight when booking for antenatal care, maternal height, maternal age, birthweight, gestation at birth and infant gender. Variance inflation factors were used to assess for multicollinearity within all multiple logistic regression models. The results indicated potential multicollinearity of marital status and other measures of deprivation due to moderately high variance inflation factors at all timepoints. Marital status was therefore omitted from all of the final multiple logistic regression models. Variance inflation factors were low (<2) between all other independent variables. Additionally, each model was assessed for a linear relationship between the continuous independent variables and the logit transformation of the dependent variable using the Box–Tidwell test for linearity. Where the assumption for linearity was not met, higher ordinal terms were included within the model.

The multiple logistic regression model for each separate timepoint was assessed using the Hosmer–Lemeshow goodness of fit test to determine how well the data fit the model. The presence of outliers or points of leverage was explored using Cook's distance and the studentized residuals.

### 2.4 Ethical statement

Ethical approval was obtained for this project (IRAS project number 207998).

## 3 RESULTS

Of the 1301 women with a BMI ≥ 35 kg/m² attending for antenatal care and giving birth to a live infant within the Trust from 2009 to 2015, 1146 (88.1%) had at least one child measurement available. Measurements were available for 91.6% of those attending their first antenatal appointment from 2009 to summer 2012. However, measurements were only available for 81.2% of those attending their first antenatal appointment from summer 2012 to 2015 as only 17 of these children had reached school age when the data was obtained from SystmOne. The average age of weight was 47.7 (±11.9) days at 6–8 weeks, 9.4 (±1.3) months at 9–12 months and 4.7 (±0.31) years at school entry.

**Figure 1:**
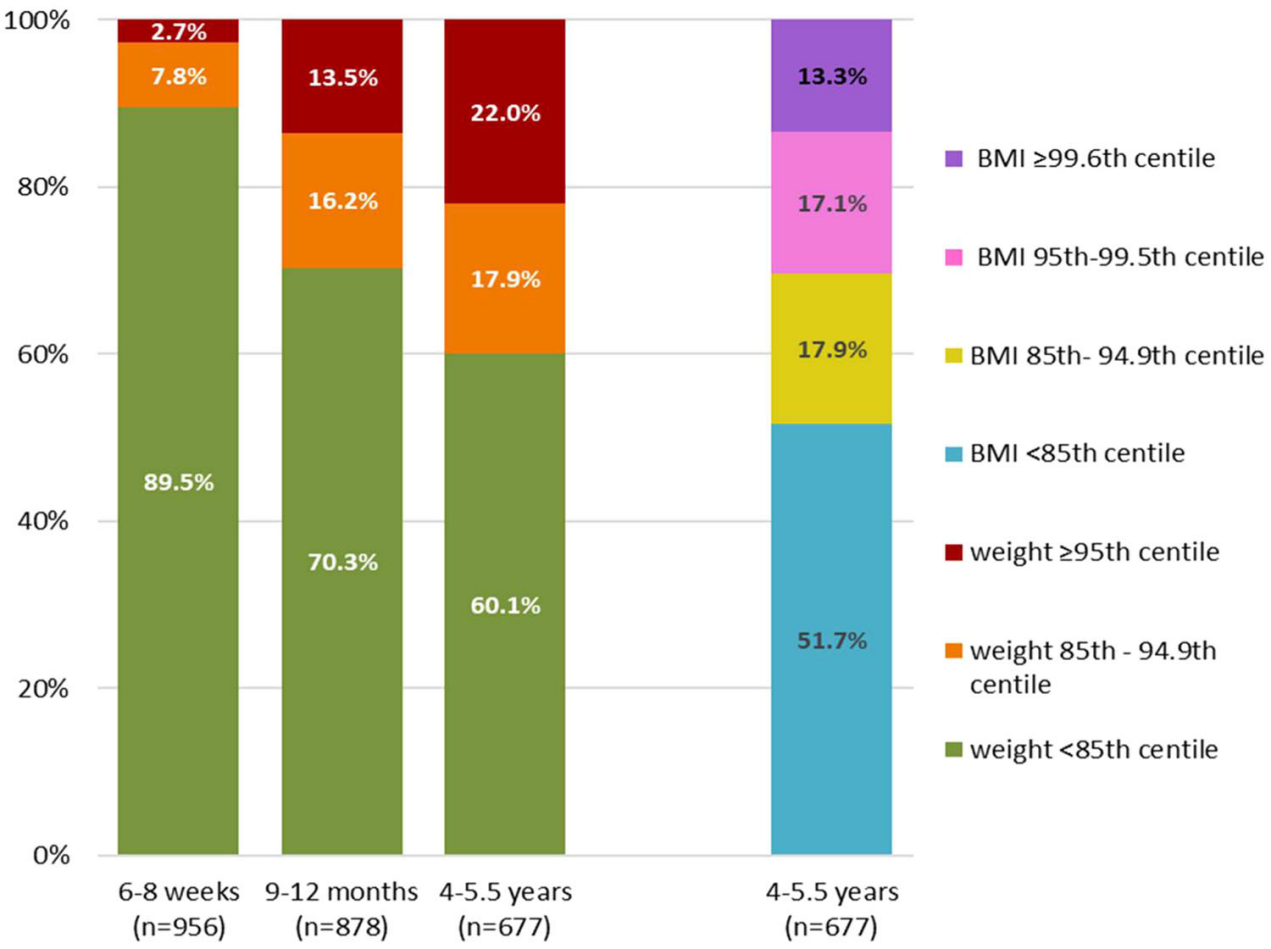
Classification of weight centiles at 6–8 weeks, 9–12 months and weight and BMI centiles at school entry for children born to mothers with BMI ≥ 35. BMI, body mass index.

Height was poorly recorded before school age, therefore overweight and obesity were classified using weight centiles only at 6–8 weeks and 9–12 months. Figure 1 shows the proportion of children at each age who were classified as overweight or obese. The proportion of children with weight ≥ 95th centile increased with age, being just 2.7% of children at 6–8 weeks, but 22.0% of children by school entry. When height was also taken into account to calculate child BMI at school entry, the proportion of children with obesity was 30.4%, with 13.3% of school‐age children having a BMI ≥ 99.6th centile. Of those with a weight ≥85th centile at 6–8 weeks and 9–12 months 33.0% and 19.5%, respectively, had been born LGA. By school age, only 15.6% of those with overweight or obesity had been born LGA.

### 3.1 Univariate analysis

Of the 1146 women with at least one child weight available, 79.7% had attended the antenatal healthy lifestyle service and 20.3% of women had not attended. Table 1 provides the crude odds of weight ≥ 95th centile at 6–8 weeks and 9–12 months and of childhood BMI ≥95th centile and ≥99.6th centile at school entry and Table 2 the crude odds of weight ≥85th centile at 6–8 weeks and 9–12 months and of childhood BMI ≥85th centile at school entry according to uptake of the antenatal healthy lifestyle service, GWG and other sociodemographic characteristics.

**Table 1:** Crude ORs and 95% CIs for childhood obesity according to the uptake of the antenatal healthy lifestyle service, gestational weight gain and sociodemographic characteristics.

	Weight ≥ 95th centile at 6–8 weeks (*n *= 26/956), OR (95% CI)	*p* Value	Weight ≥ 95th centile at 9–12 months (*n *= 119/878), OR (95% CI)	*p* Value	BMI ≥ 95th centile at school entry (*n* = 206/677), OR (95% CI)	*p* Value	BMI ≥ 99.6th centile at school entry (*n *= 90/677), OR (95% CI)	*p* Value
Number of healthy lifestyle sessions attended								
Not attended	REF		REF		REF		REF	
Attended	1.703 (0.505, 5.736)	0.391	0.943 (0.582, 1.528)	0.811	0.750 (0.524, 1.074)	0.116	1.120 (0.675, 1.859)	0.662
Weight gain according to IOM	(*n* = 838)		(*n* = 771)		(*n* = 601)		(*n* = 601)	
Less than recommended	0.482 (0.134, 1.728)	0.262	1.036 (0.601, 1.787)	0.898	1.252 (0.790, 1.984)	0.339	1.030 (0.533, 1.989)	0.931
Recommended	REF		REF		REF		REF	
More than recommended	1.940 (0.734, 5.131)	0.182	1.565 (0.937, 2.614)	0.087	1.723 (1.106, 2.684)	0.016*	1.778 (0.976, 3.241)	0.060
Parity								
1	REF		REF		REF		REF	
2	1.605 (0.656, 3.925)	0.300	1.205 (0.749, 1.941)	0.442	0.745 (0.500, 1.110)	0.148	0.699 (0.403, 1.215)	0.205
3+	0.659 (0.213, 2.036)	0.468	1.287 (0.799, 2.075)	0.300	0.857 (0.576, 1.277)	0.448	0.951 (0.562, 1.610)	0.851
Deprivation								
Most deprived quintile	REF		REF		REF		REF	
Second most deprived quintile	0.293 (0.066, 1.293)	0.105	0.838 (0.516, 1.362)	0.476	0.996 (0.671, 1.480)	0.985	0.985 (0.578, 1.677)	0.955
Middle quintile	1.078 (0.351, 3.308)	0.896	0.815 (0.440, 1.510)	0.516	0.892 (0.531, 1.498)	0.666	0.916 (0.455, 1.847)	0.807
Least deprived two quintiles	1.347 (0.480, 3.782)	0.571	0.654 (0.333, 1.284)	0.217	0.491 (0.263, 0.916)	0.025*	0.536 (0.221, 1.302)	0.169
Highest occupation	(*n* = 930)		(*n* = 856)		(*n* = 656)		(*n* = 656)	
Managerial and professional occupations	1.340 (0.461, 3.896)	0.591	0.534 (0.287, 0.994)	0.048*	0.619 (0.351, 1.094)	0.099	0.779 (0.349, 1.737)	0.542
Intermediate occupations	1.092 (0.389, 3.063)	0.868	0.706 (0.418, 1.194)	0.194	0.889 (0.548, 1.443)	0.634	1.167 (0.600, 2.270)	0.649
Routine and manual occupations	0.394 (0.114, 1.362)	0.141	0.532 (0.317, 0.891)	0.017*	1.088 (0.693, 1.708)	0.715	1.241 (0.663, 2.323)	0.500
Housewife/unemployed/student	REF		REF		REF		REF	
Maternal education	(*n* = 339)		(*n* = 302)		(*n* = 184)		(*n* = 184)	
A'level/equivalent or lower	REF		REF		REF		REF	
Degree or above	1.979 (0.462, 8.471)	0.357	0.676 (0.266, 1.716)	0.410	0.758 (0.350, 1.640)	0.481	0.960 (0.332, 2.774)	0.940
Marital status	(*n* = 951)		(*n* = 874)		(*n* = 676)		(*n* = 676)	
Married/civil partner	0.594 (0.199, 1.778)	0.352	0.576 (0.316, 1.050)	0.072	0.585 (0.333, 1.030)	0.063	0.614 (0.300, 1.257)	0.182
Partner	0.459 (0.156, 1.350)	0.157	0.543 (0.305, 0.967)	0.038*	0.637 (0.373, 1.088)	0.098	0.593 (0.301, 1.169)	0.132
Single	REF		REF		REF		REF	
Smoker at first antenatal appointment	(*n* = 955)	0.115	(*n* = 877)	0.330	(*n* = 676)	0.033*	(*n* = 676)	0.003**
	0.312 (0.073, 1.330)		1.250 (0.798, 1.958)		1.507 (1.034, 2.197)		2.077 (1.291, 3.340)	
Ethnicity	(*n* = 951)		(*n* = 872)		*N* = 674		(*n* = 674)	
Not W/B	0.988 (0.130, 7.494)^a^	0.991	1.346 (0.503, 3.599)	0.554	1.484 (0.706, 3.116)	0.298	0.960 (0.328, 2.810)	0.940
Breastfeeding initiation	(*n* = 941)	0.831	(*n* = 858)	0.184	(*n* = 662)	0.930	(*n* = 662)	0.417
Yes	0.918 (0.420, 2.007)		1.309 (0.880, 1.947)		1.015 (0.728, 1.415)		0.830 (0.528, 1.303)	
Breastfed at hospital discharge	(*n* = 923)	0.439	(*n* = 845)	0.748	(*n* = 659)	0.909	(*n* = 659)	0.465
Yes	1.361 (0.624, 2.968)		1.067 (0.720, 1.580)		0.981 (0.701, 1.372)		0.845 (0.537, 1.329)	
Caesarean section birth	(*n* = 955)	0.997	*n* = 877)	0.372	(*n* = 676)	0.697	(*n* = 676)	0.414
	1.001 (0.449, 2.231)		1.196 (0.808, 1.771)		0.934 (0.662, 1.318)		1.210 (0.766, 1.910)	
Maternal diabetes (GDM or pre‐existing)	(*n* = 840)	0.938	(*n* = 769)	0.622	(*n* = 587)	0.535	(*n* = 587)	0.433
	0.957 (0.322, 2.844)		0.870 (0.499, 1.516)		1.166 (0.781, 1.892)		1.286 (0.686, 2.411)	
Pregnancy‐induced hypertension/pre‐eclampsia	(*n* = 882)	0.988	(*n* = 812)	0.077	(*n* = 638)	0.729	(*n* = 638)	0.935
	0.991 (0.288, 3.413)		0.526 (0.258, 1.073)		0.918 (0.566, 1.488)		0.973 (0.506, 1.874)	

Abbreviations: BMI, body mass index; CI, confidence interval; GDM, gestational diabetes; IOM, Institute of Medicine; OR, odds ratio; REF, referent category; W/B, White/British.

^a^ Single case only in one category.

* *p* < 0.05

** *p* < 0.01.

**Table 2:** Crude ORs and 95% CIs for childhood overweight according to the uptake of the antenatal healthy lifestyle service, gestational weight gain and sociodemographic characteristics.

	Weight ≥85th centile at 6–8 weeks (*n* = 100/956), OR (95% CI)	*p* Value	Weight ≥85th centile at 9–12 months (*n* = 261/878), OR (95% CI)	*p* Value	BMI ≥85th centile school entry (*n* = 327/677), OR (95% CI)	*p* Value
Number of healthy lifestyle sessions attended						
Not attended	REF		REF		REF	
Attended	1.170 (0.667, 2.053)	0.584	0.888 (0.618, 1.274)	0.517	0.762 (0.543, 1.068)	0.115
Weight gain according to IOM	(*n* = 838)		(*n* = 771)		(*n* = 601)	
Less than recommended	1.405 (0.566, 1.929)	0.888	0.752 (0.505, 1.119)	0.160	1.507 (1.001, 2.269)	0.049*
Recommended	REF		REF		REF	
More than recommended	2.319 (1.323, 4.063)	0.003**	1.360 (0.932, 1.983)	0.111	1.839 (1.229, 2.752)	0.003**
Parity						
1	REF		REF		REF	
2	1.824 (1.115, 2.985)	0.017*	0.913 (0.642, 1.296)	0.610	0.957 (0.664, 1.380)	0.815
3+	0.863 (0.485, 1.533)	0.614	1.005 (0.707, 1.430)	0.976	0.781 (0.538, 1.133)	0.193
Deprivation						
Most deprived quintile	REF		REF		REF	
Second most deprived quintile	0.633 (0.364, 1.099)	0.104	1.035 (0.722, 1.483)	0.853	1.089 (0.752, 1.576)	0.653
Middle quintile	0.700 (0.356, 1.375)	0.301	0.861 (0.541, 1.369)	0.527	0.826 (0.512, 1.332)	0.433
Least deprived two quintiles	0.625 (0.310, 1.259)	0.189	1.049 (0.665, 1.657)	0.836	0.542 (0.322, 0.912)	0.021*
Highest occupation	(*n* = 930)		(*n* = 856)		(*n* = 656)	
Managerial and professional occupations	0.873 (0.430, 1.773)	0.707	0.911 (0.570, 1.456)	0.696	0.759 (0.462, 1.248)	0.277
Intermediate occupations	1.635 (0.917, 2.917)	0.096	1.192 (0.785, 1.809)	0.411	1.043 (0.669, 1.627)	0.851
Routine and manual occupations	1.005 (0.559, 1.806)	0.988	0.861 (0.575, 1.290)	0.468	0.964 (0.633.1.468)	0.864
Housewife/unemployed/student	REF		REF		REF	
Maternal education	(*n* = 339)		(*n* = 302)		(*n* = 184)	
A'Level/equivalent or lower	REF		REF		REF	
Degree or above	0.794 (0.313, 2.016)	0.627	0.681 (0.360, 1.288)	0.237	0.850 (0.428, 1.689)	0.642
Marital status	(*n* = 951)		*(n* = 874)		(*n* = 676)	
Married/civil partner	0.526 (0.283, 0.976)	0.042*	0.768 (0.473, 1.248)	0.287	0.671 (0.388, 1.160)	0.153
Partner	0.525 (0.292, 0.945)	0.032*	0.644 (0.402, 1.030)	0.066	0.701 (0.415, 1.183)	0.184
Single	REF		REF		REF	
Smoker at first antenatal appointment	(*n* = 955)	0.477	(*n* = 877)	0.572	(*n* = 676)	0.580
	0.823 (0.482, 1.407)		1.105 (0.781, 1.564)		1.107 (0.773, 1.584)	
Ethnicity	(*n* = 951)		(*n* = 872)		(*n* = 674)	
Not W/B	2.060 (0.880, 4.820)	0.096	1.263 (0.579, 2.754)	0.558	1.321 (0.640, 2.724)	0.451
Breastfeeding initiation	(*n* = 941)	0.469	(*n* = 858)	0.057	(*n* = 662)	0.398
Yes	1.172 (0.763, 1.800)		1.334 (0.992, 1.796)		1.141 (0.840, 1.549)	
Breastfed at hospital discharge	(*n* = 923)	0.298	(*n *= 845)	0.166	(*n* = 659)	0.884
Yes	1.249 (0.822, 1.897)		1.232 (0.917, 1.656)		0.977 (0.718, 1.330)	
Caesarean birth	(*n* = 955)	0.756	(*n* = 877)	0.154	(*n* = 676)	0.835
	0.934 (0.609, 1.434)		1.240 (0.923, 1.667)		0.967 (0.705, 1.327)	
Maternal diabetes (GDM or pre‐existing)	(*n* = 840)	0.123	(*n* = 769)	0.915	(*n* = 587)	0.768
	0.597 (0.310, 1.150)		1.022 (0.687, 1.521)		1.071 (0.680, 1.684)	
Pregnancy‐induced hypertension/pre‐eclampsia	(*n* = 882)	0.742	(*n* = 812)	0.259	(*n* = 638)	0.581
	1.107 (0.605, 2.025)		0.771 (0.492, 1.210)		0.883 (0.568, 1.373)	

Abbreviations: BMI, body mass index; CI, confidence interval; GDM, gestational diabetes; IOM, Institute of Medicine; OR, odds ratio; REF, referent category; W/B, White/British.

* *p* < 0.05

** *p* < 0.01.

There was no difference in the odds of childhood overweight or obesity at any of the timepoints with healthy lifestyle attendance compared to no attendance at the service. Infants of women with excessive GWG according to IOM recommendations had higher odds of overweight at 6–8 weeks and of overweight or obesity at school entry. There was also a trend for increased childhood obesity at 9–12 months and severe obesity at school entry with excessive GWG. However, at 6–8 weeks, only 58.3% of infants with obesity were born to mothers with excessive GWG, and at 9–12 months and school age, less than 45% of children with obesity were born to mothers with excessive GWG.

At school entry childhood overweight and obesity decreased with lower levels of deprivation. Additionally, at 9–12 months, childhood obesity decreased with higher household occupations. At several timepoints, the odds of childhood overweight or obesity were also lower for women who were not single when registering for antenatal care. The infants of women who smoked when attending their first antenatal appointment had higher odds of obesity and severe obesity at school entry. Maternal ethnicity, maternal education, breastfeeding initiation or breastfeeding at discharge from the maternity unit, caesarean birth, maternal diabetes and pregnancy‐induced hypertension or pre‐eclampsia did not significantly increase the odds of childhood overweight or obesity at any timepoint in this cohort of women with a BMI ≥ 35 kg/m^2^.

### 3.2 Multiple logistic regression analysis

Multiple logistic regression models for factors associated at the different timepoints with childhood obesity (Table 3) and overweight (Table 4) are provided.

**Table 3:** Multiple logistic regression models of factors associated with childhood obesity (≥95th centile) at each of the different timepoints and severe obesity (≥99.6th centile) at school entry.

	Weight ≥ 95th centile at 6–8 weeks, Nagelkerke *R* ^2^ = 0.501, *χ* ^2^ = 101.3, *p* < 0.001, *n* = 813		Weight ≥ 95th centile at 9–12 months, Nagelkerke *R* ^2^ = 0.148, *χ* ^2^ = 64.4, *p* < 0.001, *n* = 749		BMI ≥ 95th centile at school entry, Nagelkerke *R* ^2^ = 0.113, *χ* ^2^ = 48.0, *p* < 0.001, *n* = 583		BMI ≥ 99.6th centile at school entry, Nagelkerke *R* ^2^ = 0.133, *χ* ^2^ = 42.9, *p* < 0.001, *n* = 583	
Predictor	aOR (95% CI)^a^	*p* Value	aOR (95% CI)^a^	*p* Value	aOR (95% CI)^a^^,^ b	*p* value	aOR (95% CI)^a^	*p* Value
Weight gain according to IOM								
Less than recommended	0.395 (0.082, 1.905)	0.247	1.001 (0.554, 1.807)	0.998	1.350 (0.820, 2.223)	0.239	0.938 (0.460, 1.913)	0.861
Recommended	REF		REF		REF		REF	
More than recommended	0.598 (0.168, 2.131)	0.428	1.119 (0.636, 1.967)	0.696	1.640 (1.012, 2.656)	0.044*	1.726 (0.900, 3.312)	0.101
Parity								
1	REF		REF		REF		REF	
2	2.264 (0.621, 8.254)	0.216	1.071 (0.620, 1.852)	0.805	0.659 (0.411, 1.057)	0.084	0.685 (0.356, 1.319)	0.258
3+	1.021 (0.189, 5.505)	0.981	1.045 (0.553, 1.973)	0.893	0.682 (0.396, 1.174)	0.168	0.740 (0.362, 1.513)	0.409
Deprivation								
Most deprived quintile	REF		REF		REF		REF	
Second most deprived quintile	0.177 (0.028, 1.132)	0.067	0.916 (0.524, 1.599)	0.757	1.363 (0.865, 2.147)	0.182	1.044 (0.561, 1.941)	0.892
Middle quintile	0.769 (0.167, 3.533)	0.736	0.965 (0.488, 1.907)	0.918	1.127 (0.606, 2.095)	0.706	0.997 (0.432, 2.299)	0.994
Least deprived two quintiles	1.599 (0.364, 7.023)	0.534	0.721 (0.322, 1.611)	0.425	0.650 (0.314, 1.345)	0.246	0.507 (0.164, 1.560)	0.236
Occupation								
Higher managerial or professional	0.453 (0.083, 2.480)	0.361	0.451 (0.209, 0.970)	0.042*	0.410 (0.200, 0.841)	0.015*	0.540 (0.202, 1.443)	0.219
Intermediate occupations	0.389 (0.082, 1.844)	0.235	0.649 (0.348, 1.212)	0.175	0.759 (0.432, 1.331)	0.336	0.979 (0.452, 2.118)	0.957
Routine or manual	0.180 (0.033, 0.972)	0.046*	0.547 (0.304, 0.985)	0.044*	0.931 (0.557, 1.556)	0.785	1.045 (0.514, 2.128)	0.902
Unemployed/housewife	REF		REF		REF		REF	
Smoking	0.685 (0.090, 5.228)	0.715	1.384 (0.805, 2.380)	0.240	1.554 (0.973, 2.482)	0.055	2.695 (1.448, 5.018)	0.002**

Abbreviations: aOR, adjusted odds ratio; BMI, body mass index; CI, confidence interval; IOM, Institute of Medicine; REF, referent category.

^a^ Adjusted for maternal weight when booking for pregnancy, maternal height, maternal age, birthweight, gestation at birth and infant gender.

^b^ Additionally adjusted for birthweight squared.

* *p* < 0.05

** *p* < 0.01.

**Table 4:** Multiple logistic regression models of factors significantly associated with childhood overweight (≥85th centile) at each of the different timepoints.

	Weight ≥ 85th centile at 6–8 weeks, Nagelkerke *R* ^2^ = 0.456, *χ* ^2^ = 215.6, *p* < 0.001, *n* = 813		Weight ≥ 85th centile at 9–12 months, Nagelkerke *R* ^2^ = 0.165, *χ* ^2^ = 92.9, *p* < 0.001, *n* = 749		BMI ≥ 85th centile school entry, Nagelkerke *R* ^2^ = 0.134, *χ* ^2^ = 61.5, *p* < 0.001, *n* = 583	
Predictor	aOR (95% CI)^a^	*p* Value	aOR (95% CI)^a^	*p* Value	aOR (95% CI)^a^^,^ b	*p* Value
Weight gain according to IOM						
Less than recommended	1.432 (0.656, 3.127)	0.367	0.730 (0.471, 1.131)	0.159	1.815 (1.163, 2.832)	0.009**
Recommended	REF		REF		REF	
More than recommended	1.783 (0.880, 3.609)	0.108	0.987 (0.649, 1.501)	0.951	1.651 (1.062, 2.567)	0.026*
Parity						
1	REF		REF		REF	
2	1.906 (0.970, 3.744)	0.061	0.866 (0.571, 1.312)	0.496	0.758 (0.492, 1.166)	0.207
3+	0.911 (0.384, 2.164)	0.833	0.956 (0.590, 1.550)	0.855	0.526 (0.317, 0.871)	0.013*
Deprivation						
Most deprived quintile	REF		REF		REF	
Second most deprived quintile	0.527 (0.259, 1.073)	0.077	1.074 (0.702, 1.641)	0.743	1.303 (0.851, 1.995)	0.223
Middle quintile	0.484 (0.192, 1.224)	0.125	0.935 (0.549, 1.595)	0.806	0.887 (0.504, 1.562)	0.679
Least deprived two quintiles	0.757 (0.300, 1.913)	0.557	1.285 (0.742, 2.224)	0.371	0.643 (0.349, 1.186)	0.157
Occupation						
Higher managerial or professional	0.410 (0.147, 1.140)	0.087	0.893 (0.492, 1.621)	0.711	0.577 (0.307, 1.082)	0.087
Intermediate occupations	1.072 (0.493, 2.333)	0.861	1.181 (0.714, 1.953)	0.517	0.828 (0.490, 1.399)	0.482
Routine or manual	0.809 (0.391, 1.674)	0.569	0.917 (0.569, 1.477)	0.720	0.766 (0.470, 1.249)	0.286
Unemployed/housewife	REF		REF		REF	
Smoking	1.701 (0.846, 3.421)	0.136	1.375 (0.896, 2.111)	0.145	1.217 (0.782, 1.893)	0.384

Abbreviations: aOR, adjusted odds ratio; BMI, body mass index; CI, confidence interval; IOM, Institute of Medicine; REF, referent category.

^a^ Adjusted for maternal weight when booking for pregnancy, maternal height, maternal age, birthweight, gestation at birth and infant gender.

^b^ Additionally adjusted for birthweight squared.

* *p* < 0.05

** *p* < 0.01.

Once adjusting for other factors within the analysis, deprivation no longer added significantly to any of the models. Excessive GWG continued to increase the odds of obesity at school entry once adjusting for other factors. GWG outside of the recommended range, both inadequate and excessive, also increased the risk of overweight at school entry. Being a smoker at the first antenatal appointment no longer reached significance for increased odds of obesity at school entry but remained significant in the multiple logistic regression model for severe obesity at school entry.

Outliers or extreme points of leverage were only noted within the models at 6‐8 weeks gestation. The multiple logistic regression model fitted less well at this timepoint for women gaining weight in accordance with IOM recommendations or below IOM recommendations due to the limited number of cases of children with obesity within these categories at this point in time.

## 4 DISCUSSION

Prevalence of children with obesity (BMI ≥ 95th centile) by school entry was high in this cohort at 30.4%, with almost half of children (48.3%) having either overweight or obesity by school entry. The proportion of children with obesity at school entry was high compared to both the national average of 9.7% and the local prevalence of 11.4% in 2018–2019 (NHS Digital, 2019b). The proportion of children with severe obesity (BMI ≥ 99.6th centile) at 13.3% was far higher than the national average of 2.4% and the local prevalence of 3.1% for 2018–2019 (NHS Digital, 2019b). Indeed, within this cohort of women with a BMI ≥ 35 kg/m^2^, there were more children with severe obesity at school entry than there were children with obesity at school entry (BMI ≥ 95th centile) in the local area when considering women of all BMI categories. Even when looking specifically at women with obesity the figures within this cohort were high, as nationally 26% of children born to mothers with obesity were themselves obese at school entry, and a further 16% were overweight (NHS Digital, 2019c). This may in part be due to this study only including women with class 2 or class 3 obesity as all had a BMI ≥ 35 kg/m². However, it clearly shows an association between maternal early pregnancy BMI and childhood weight outcomes up to 5 years later, with increasing maternal weight at the start of pregnancy associated with an increased risk of childhood severe obesity at school age. The noticeably higher prevalence of childhood overweight and obesity within women with the most severe forms of obesity is of concern given the rising prevalence of obesity within the United Kingdom. Furthermore, the potential importance of assessing outcomes separately according to a class of obesity is highlighted.

Compared to GWG within the recommended range, excessive weight gain (above IOM recommendations) was seen within the multiple logistic regression analysis within this study to be associated with increased odds of childhood overweight and obesity at school entry. Much other research has also looked at the long‐term association between GWG and offspring obesity (Lau et al., 2014; Sridhar et al., 2014; Tie et al., 2014; Voerman et al., 2019). One systematic review found each additional 1 kg of GWG increased the child's BMI *z*‐score by between 0.006 and 0.06 units and elevated the risk of overweight or obesity by 1%–23% after adjusting for confounders (Lau et al., 2014). Further studies have also shown exceeding IOM guidelines to be associated with a 46% increase in odds of childhood overweight/obesity at age 2–5 years after adjusting for multiple confounding factors (Sridhar et al., 2014) and to increase the odds of childhood overweight or obesity from age 2–18 years (adjusted OR: 1.33 [95% CI: 1.18–1.50]) (Tie et al., 2014). The most recent individual participant analysis has similarly shown excessive GWG to increase early childhood obesity (age 2–5 years), mid‐childhood obesity (5–10 years) and late childhood obesity (10–18 years) (Voerman et al., 2019). There is some disagreement over which maternal BMIs show the most evident effect of excessive GWG on childhood weight. An American cohort suggested the most notable effect was among women with a prepregnancy BMI in the recommended range (Sridhar et al., 2014), but the individual participant analysis saw the largest effects in women with prepregnancy obesity gaining excessive weight gain (Tie et al., 2014). This lack of clarity may in part be due to the inherent limitations of exploring the association between child weight and GWG given the issues with GWG measurement, especially regarding the timing of weighing, and the potential for unmeasured confounding factors such as familial characteristics to influence the results (Lau et al., 2014). However, the important impact of maternal health and diet both before and during pregnancy on long‐term offspring health and development through the role of epigenetics is known to be important and requires continued focus to obtain optimal long‐term childhood outcomes (Aldhous et al., 2018; Lorite Mingot et al., 2017).

Little has been done to date to evaluate the association between pregnancy lifestyle interventions and long‐term infant health. Two systematic reviews of randomised controlled trial (RCT), quasi‐randomised or cluster randomised study evidence (Dalrymple et al., 2018; Raab et al., 2021), found only five studies that evaluated childhood anthropometric outcomes up to 5 years of age (Chiavaroli et al., 2018; Dodd et al., 2020; Grotenfelt et al., 2020; Kolu et al., 2016; Ronnberg et al., 2017). Three of these studies recruited very few children born to women with obesity during pregnancy. Only 16% of the infants within a Swedish RCT were noted to have had mothers with obesity (Ronnberg et al., 2017) and within a Finish RCT, the two women with a BMI ≥ 40 kg/m^2^ were excluded from the analysis due to being outliers (Kolu et al., 2016; Luoto et al., 2011). Within the New Zealand trial women had a mean BMI of 25.4 ± 2.9 kg/m^2^ in the control group and of 25.5 ± 4.3 kg/m^2^ in the exercise group, with no women in the control group having a BMI ≥ 35 kg/m^2^ and the maximum BMI in the exercise group being 37.1 kg/m^2^ (Chiavaroli et al., 2018; Hopkins et al., 2010). The other two studies looking at the long‐term influence of antenatal interventions either exclusively focussed on women with overweight or obesity (Dodd et al., 2020) or recruited a sizable sample of women with obesity, with 294 of the 493 pregnant women recruited having a BMI ≥ 30 kg/m^2^ (Grotenfelt et al., 2020; Rönö et al., 2014). Within this current study, attendance at the midwife‐led antenatal healthy lifestyle service was not associated with childhood overweight or obesity at any timepoint within the univariate analyses. While the previous research in this area provides limited evidence surrounding interventions in women with a raised BMI, they similarly showed no statistically significant differences in child growth at 3–5 years (Dodd et al., 2020), at 5 years (Grotenfelt et al., 2020; Ronnberg et al., 2017) or at 7 years (Chiavaroli et al., 2018; Kolu et al., 2016) from a lifestyle intervention during pregnancy. Of interest, is that while two of the above studies showed no differences in child BMI, the infants of mothers who received the intervention compared to those who received the control, were shown to have worse metabolic health especially related to lipid metabolism at 5 years (Grotenfelt et al., 2020) and significantly increased body fat and abdominal adiposity at 7 years of age (Chiavaroli et al., 2018). The reason for these differences was unclear within both studies. The lack of association between childhood weight and attendance at the midwife‐led antenatal healthy lifestyle service within this study, as well as in previous RCTs with long‐term follow up could potentially be due to the limited impact of the interventions on GWG. GWG did not differ in women attending the antenatal healthy lifestyle service (Fair & Soltani, 2024), and was only significantly different between control and intervention groups within one of the RCTs (Ronnberg et al., 2017) and even then GWG was only reduced by 1.1 kg. Therefore, further establishment of interventions that are effective at reducing GWG and enhancing clinical outcomes in women with obesity is warranted. Given that the association between long‐term child health and intervention, along with other maternal factors, was similar within this matched data to the results from RCTs, further use of matched cohort data is suggested as a more cost‐effective solution for intervention follow up than expensive RCTs.

While 33% of children classified as overweight or obese at 6–8 weeks had been born LGA, this proportion had dropped to only 15.6% of children who were overweight or obese at school entry who had been born LGA. The literature from several cohort studies has noted that children born LGA are more likely to be overweight or obese at 6–12 months (Moschonis et al., 2008) and at 4–6 years old (Kaul et al., 2019). Within the second cohort study, there was a 39.4% increase in overweight or obesity in children born LGA; with LGA noted to have a larger impact than maternal diabetes during pregnancy (Kaul et al., 2019). However, maternal BMI or weight status was not considered as a confounder within that study (Kaul et al., 2019) and the other cohort study only classified women as overweight or not overweight in pregnancy without considering women with obesity as a separate subcategory (Moschonis et al., 2008). The literature has also shown high infant birthweight to be associated with childhood overweight up to 2 years of age in a meta‐analysis of prospective studies (Weng et al., 2012) and to be a predictor of overweight/obesity at school age (Apfelbacher et al., 2008). However, again the second of these studies did not consider the potential impact of maternal BMI or weight status by adjusting for this factor within the analysis (Apfelbacher et al., 2008). The independent risk of being born LGA to a woman with prepregnancy obesity therefore remains unclear.

Caesarean birth was not linked to childhood obesity in this sample of women with raised BMI. Numerous previous studies and reviews have shown Caesarean birth to be linked to an increased risk of overweight or obesity up to school age (Kaul et al., 2019; Keag et al., 2018). However, limitations of previous research have been noted especially around the lack of adjustment for maternal BMI (Masukume et al., 2019). A British study that carefully adjusted for maternal prepregnancy BMI showed no association between mode of birth and childhood overweight and therefore hypothesised that the previously noted link was likely to be mediated by the additional risk of giving birth by Caesarean with a raised maternal BMI (Masukume et al., 2019). An additional review of prenatal factors that predict later childhood obesity found Caesarean birth may influence childhood obesity (Liao et al., 2019); however, they noted being born by Caesarean is also linked with antibiotic exposure and poor early breastfeeding, both of which are other factors known to be associated with childhood obesity.

Once controlling for other factors within the multiple logistic regression analysis, infants of women who smoked at the first antenatal appointment had higher odds of severe obesity at school entry, as well as a trend towards increased obesity at school entry, but not at earlier timepoints. Several meta‐analyses have also previously identified that infants of mothers who smoked during pregnancy are at higher risk of overweight and obesity during childhood (Riedel et al., 2014; Weng et al., 2012). Within one previous meta‐analysis, the effect of higher childhood obesity in children of women who smoked during pregnancy remained after excluding studies that did not adjust for potential confounders including maternal BMI, parental education and birthweight (Riedel et al., 2014). A further systematic review of prediction models of childhood overweight or obesity from 1 to 13 years also found smoking during pregnancy to be significantly associated with overweight and obesity within four of the eight included models (Ziauddeen et al., 2018). The association between smoking and child weight may only be evident by school entry within this study as smoking during pregnancy is known to increase the risk of having infants of low birthweight (Inoue et al., 2017). There may therefore be a lag before seeing an association between smoking during pregnancy and childhood obesity, as the infant has first to overcome the initial growth restriction during pregnancy.

There was also a relationship between childhood weight and socioeconomic status within this study. It was noted that lower deprivation levels were no longer significantly associated with lower levels of childhood overweight or obesity at school entry once controlling for other factors, including household occupation. However, being in a household where no‐one was in employment increased childhood obesity at different timepoints. Others have also previously noted the importance of sociodemographic factors. An American study has shown the highest prevalence of overweight (BMI ≥ 85th centile) in elementary school among those of low socioeconomic status, although the potentially confounding effects of maternal BMI or birthweight were not considered within this study (Moreno et al., 2013). A further Canadian cohort found childhood overweight and obesity decreased at age 4–6 years with increasing household income (Kaul et al., 2019). The systematic review of prediction models of childhood overweight or obesity from 1 to 13 years also found that sociodemographic factors such as marital status, paternal and maternal education, paternal income, maternal occupation and ethnicity were included within different models, although each factor was only present within one of the eight included models (Ziauddeen et al., 2018). However, it clearly shows the importance of developing interventions that do not just focus on the mother as an individual during pregnancy, but on wider social determinants of health. Consideration should be given to utilising a socioecological framework when developing future interventions that incorporates not only the woman, but other influences including her family and home, work and peers, community, industry and government and culture and society, as well as the interaction between these aspects (Hill, 2021). Additionally, given the large proportion of children with overweight and obesity born to women with a raised pregnancy BMI demonstrated within this study, the importance for long‐term child health of addressing maternal weight before pregnancy is highlighted.

### 4.1 Strengths and limitations

This cohort study explored the association between an antenatal healthy lifestyle service and child weight among a large number of women with a BMI ≥ 35 kg/m^2^, a category often lacking in previous research. Additionally, it is one of the few studies taking advantage of data linkage to investigate long‐term infant weight outcomes. Some limitations however need to be acknowledged. *p* values were not corrected for multiple hypothesis testing within the analysis. It is acknowledged that the large number of statistical tests performed increases the risk of a type I error. Some of the statistically significant findings may therefore be due to chance. There was variation within the timing of data collection for each infant at the different timepoints. However, to account for this weight and BMI were converted into age‐appropriate centiles within the analysis. Furthermore, retrospective data collection is well known for its limitations around data collection completeness (Hasson et al., 2015). It was particularly evident within this study that education was poorly documented within the maternity notes. While factors within the analysis were identified within the literature, the retrospective nature of the study also limited the availability of some factors, for example, longer‐term breastfeeding outcomes. Additionally, childhood anthropometric data was collected within routine care and therefore recorded by various personnel, which may limit standardisation. Finally, the wider generalisability of the study is limited by the higher rate of social deprivation within the cohort than across England in general (Office for Health Improvements and Disparities, 2022b).

## 5 CONCLUSION

Matching data between two datasets was shown to be feasible using pseudoanonymised data. Current data did not suggest any association between healthy lifestyle service attendance compared to no attendance on the odds of childhood overweight or obesity up to school entry. Sociodemographic characteristics such as household occupation and maternal smoking during pregnancy were noted to be associated with long‐term childhood obesity. Future interventions need to consider how to address wider determinants of health and not just the individual woman's behaviour.

## AUTHOR CONTRIBUTIONS

Frankie J. Fair and Hora Soltani developed the protocol. Frankie J. Fair assisted with data collection. Frankie J. Fair analysed and interpreted the data. Hora Soltani supervised the analysis and interpretation of the data. Frankie J. Fair wrote the manuscript. Hora Soltani revised the manuscript. Frankie J. Fair and Hora Soltani agreed on the final manuscript.

## CONFLICT OF INTEREST STATEMENT

The authors declare no conflict of interest.

## Data Availability

The data that support the findings of this study are not publicly available within a repository as they belong to the Hospital Trusts, but the data is available from the corresponding author on reasonable request. For the purpose of open access, the author has applied a Creative Commons Attribution (CC BY) licence to any Author Accepted Manuscript version arising from this submission.
